# Causal relationship between acute pancreatitis and methylprednisolone pulse therapy for fulminant autoimmune hepatitis: a case report and review of literature

**DOI:** 10.1186/s40780-018-0111-5

**Published:** 2018-05-31

**Authors:** Daisuke Nango, Hiroki Nakashima, Yukifumi Hirose, Masaaki Shiina, Hirotoshi Echizen

**Affiliations:** 1 Departments of Pharmacy, Shin-Yurigaoka General Hospital, 255 Furusawa-tsuko, Asao-ku, Kawasaki, Kanagawa 215-0026 Japan; 2 Gastroenterology and Hepatology, Shin-Yurigaoka General Hospital, 255 Furusawa-tsuko, Asao-ku, Kawasaki, Kanagawa 215-0026 Japan; 3 Department of Pharmacotherapy, Meiji Pharmaceutical University, 2-522-1 Noshio, Kiyose, Tokyo, 204-8588 Japan

**Keywords:** Steroid pulse therapy, Autoimmune hepatitis, Acute pancreatitis, Short-term tapering

## Abstract

**Background:**

A causal relationship between acute pancreatitis and administration of glucocorticoids remains a matter of debate, since most of the reported cases were diagnosed with systemic vascular diseases (including systemic lupus erythematosus and polyarteritis nodosa) that may be responsible for the pancreatitis.

**Case presentation:**

We report a case of a 51-year-old woman who developed acute pancreatitis after receiving methylprednisolone pulse therapy for the treatment of fulminant autoimmune hepatitis (AIH). She was admitted to our hospital because of overt jaundice and back pain. Since her liver dysfunction deteriorated progressively, a liver biopsy was performed and a diagnosis of AIH was established. She was given intravenous methylprednisolone pulse therapy at 1000 mg/day for 3 days, and oral prednisolone at 40 mg/day thereafter. While her liver function improved rapidly, she started complaining of mild back pain and serum amylase and lipase levels were elevated from 5 days after the initiation of steroid therapy. A CT scan revealed mildly edematous changes around the pancreas, leading to a diagnosis of acute pancreatitis. After tapering off prednisolone, back pain disappeared, and elevated serum amylase was normalized without exacerbation of AIH. A systematic literature review identified 8 cases of acute pancreatitis developing after administration of corticosteroid pulse therapy with a median latent period of 5 days.

**Conclusions:**

The present case and reports in the literature suggest that steroid pulse therapy may cause acute pancreatitis in patients having no signs of systemic vasculitis.

## Background

Acute pancreatitis has been reported to be a rare, albeit severe, adverse reaction associated with administration of corticosteroids [1]. However, the causal relationship between corticosteroid treatment and pancreatitis remains controversial, since many reported cases were either diagnosed with systemic vasculitis [such as systemic lupus erythematosus (SLE)] that may be complicated with pancreatitis [2] or given medications known to cause pancreatitis (such as anticancer drugs) [3]. Here, we report a 51-year-old woman who developed acute pancreatitis after receiving steroid pulse therapy for the treatment of fulminant autoimmune hepatitis (AIH), which is not known to cause pancreatitis. We also discuss the causal relationship in the light of previous reports retrieved by a systematic literature survey.

## Case presentation

A well-nourished 51-year-old woman visited our hospital because of fatigue, overt jaundice, and back pain on March 2016 (day 1). In the present article, each of the important clinical events occurred during the clinical course of the patient was described by “days” after the patient’s first visit to our hospital. The patient was afebrile and had no arthralgia. Physical examination revealed icteric sclera but no signs of facial erythema. No lymphadenopathy was detected. Chest and abdominal findings were noncontributory. No bruise was observed on the abdomen. She was taking no medications. Laboratory data obtained at the first visit are shown in Table 1. Serum amylase and calcium levels were normal. The titers of antinuclear antibody and anti-mitochondrial antibody were within normal limits. IgM antibodies for hepatitis viruses Bc, A and E were negative. Her medical history included cholecystectomy due to gall bladder polyps and cholelithiasis one year earlier, uterine myoma left untreated, and undefined complaints tentatively diagnosed as autonomic imbalance. She did not smoke or drink alcohol. When she revisited for follow-up first days later, she was admitted emergently because of severely deteriorated liver function (day 5). Serum concentrations of total bilirubin, serum alanine aminotransferase, serum aspartate aminotransferase, and serum alkaline phosphatase were 17.48 mg/dL, 1099 U/L, 708 U/L, and 460 U/L, respectively. Percent prothrombin time (PT%) also decreased to 77%, indicating ongoing acute hepatic decompensation. A CT scan revealed no signs of bile duct stenosis, cholelithiasis, and neoplasms in the hepato-pancreatic region.

**Table 1:** Laboratory data of the patient at the first visit

Peripheral blood counts	Blood chemistry	Serological tests
WBC 3700 (/μL)	TP 7.6 (g/dL)	HBs antigen (−)
RBC 451 (10^4^/μL)	Alb 4.1 (g/dL)	HCV antibody (−)
Hb 13.7 (g/dL)	T-Bil 5.54 (mg/dL)	ANA ×20 (normal range < × 20)
Ht 40.7 (%)	D-Bil 4.14 (mg/dL)	IgG 2010 (mg/dL)
MCV 90 (fl)	AST 577 (U/L)	IgA 283 (mg/dL)
MCH 30.4 (pg)	ALT 1055 (U/L)	IgM 215 (mg/dL)
MCHC 33.7 (g/dL)	ALP 503 (U/L)	AMA-M2 1.6 (normal range < 7)
Platelet 8.8 (10^4^/μL)	LDH 338 (U/L)	IgM-HBc (−)
CRP 0.17 (mg/dL)	γ-GTP 241 (U/L)	IgM-HA (−)
	Amylase 82 (U/L)	IgA-HE (−)
	BUN 10.8 (mg/dL)	
	Creatinine 0.42 (mg/dL)	
	Na 140 (mEq/L)	
	K 4.1 (mEq/L)	
	Cl 106 (mEq/L)	
	Calcium 8.7 (mg/dL)	

Abbreviations: *WBC* white blood cell, *RBC* red blood cell, *Hb* hemoglobin, *Ht* hematocrit, *MCV* mean cell volume, *MCH* mean corpuscular hemoglobin, *MCHC* mean cell hemoglobin concentration, *Alb* serum albumin, *T-Bil* serum total bilirubin, *D-Bil* serum direct bilirubin, *AST* serum aspartate aminotransferase, *ALT* serum alanine aminotransferase, *ALP* serum alkaline phosphatase, *LDH* serum lactate dehydrogenase, *γ-GTP* serum γ-glutamyl transpeptidase, *BUN* blood urea nitrogen, *HBs* hepatitis B surface, *HCV* hepatitis C virus, *ANA* antinuclear antibody, *IgG* immunoglobulin G, *IgA* immunoglobulin a, *IgM* immunoglobulin M, *AMA* anti-mitochondrial antibody, *HBc* hepatitis B core, *HA* hepatitis a, *HE* hepatitis E

**Fig. 1:**
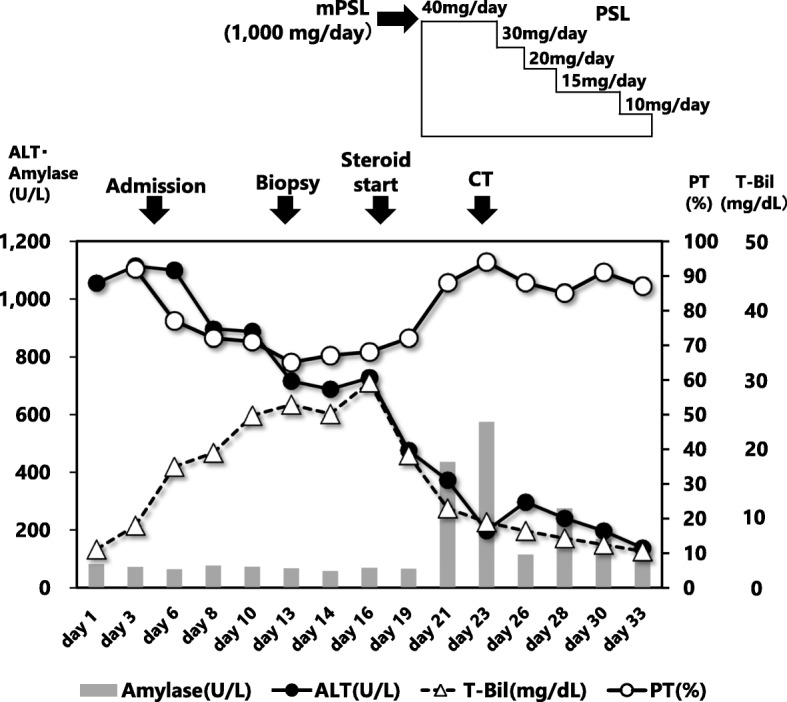
The clinical course of the present patient, with laboratory data and details of methylprednisolone pulse therapy and subsequent oral prednisolone. Each of the important clinical events occurred during the clinical course of the patient was described by “days” after the patient’s first visit to our hospital. Abbreviations: CT = computed tomography, T-Bil = serum total bilirubin, ALT = serum alanine aminotransferase, Amy = serum amylase, mPSL = methylprednisolone, PSL = prednisolone, PT = prothrombin time

Figure 1 shows the time courses of clinical events, laboratory data and medications given to the patient during her hospitalization. On the 12th day, a percutaneous fine-needle aspiration biopsy of the liver was performed. Histopathological examination revealed minimal to mild portal inflammation, mild interface hepatitis, and serious intralobular focal necrosis with plasmocytic infiltration. Inflammation was also seen around biliary canaliculi. According to the current diagnostic criteria of AIH [4, 5], the patient had an AIH score of 12 points. AIH scores of 10 to 15 points are consistent with a probable diagnosis, and scores ≥ 16 points with a definite diagnosis.

**Fig. 2:**
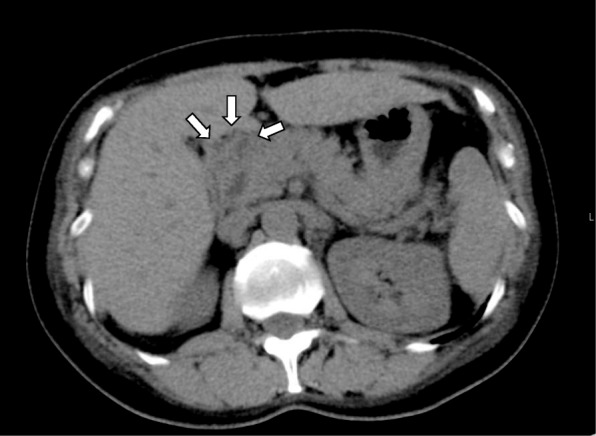
The CT image taken on Day 23 showing edematous changes around the head of pancreas (denoted by arrows). The finding is compatible with a diagnosis of acute pancreatitis

Since we judged that development of liver failure was imminent, we began corticosteroid pulse therapy with 1000 mg/day of methylprednisolone for 3 days under a diagnosis of fulminant AIH (day 17). After completion of the pulse therapy, oral prednisolone was started at 40 mg/day. Famotidine (40 mg/day) was also given to prevent gastric mucosal injury. Five days after the commencement of steroid pulse therapy (day 21), the patient developed mild back pain. And then, serum amylase and lipase levels abruptly increased to 575 U/L and 582 U/L, respectively. Since a plain CT scan revealed mild swelling around the pancreatic head (Fig. 2), a diagnosis of acute pancreatitis was made (day 23). Assuming that the development of acute pancreatitis was associated with administration of corticosteroids, we started tapering off prednisolone (day 24). Since we were concerned that an abrupt withdrawal of prednisolone might have exacerbated AIH, we underwent an accelerated tapering off of the prednisolone doses according to the schedule shown in Fig. 1. Specifically, we reduced 5 to 10 mg/day every 1 to 3 days from 40 mg/day to 10 mg/day. We monitored serum liver enzymes frequently over the following 10 days and detected no signs of reactivation of AIH. After an uneventful clinical course, the patient was discharged from hospital with a maintenance dose of prednisolone (10 mg/day) on the 33rd day after admission.

### Literature search

We searched the MEDLINE® database and the medical journal database of Japan Medical Abstracts Society for case reports describing the development of acute pancreatitis after administration of corticosteroid pulse therapy. For MEDLINE® search the following combinations of MeSH® vocabulary terms were used: “adrenal cortex hormones”[MeSH Terms] OR “steroids”[MeSH Terms]) and “pancreatitis”[MeSH Terms] AND Case Reports [ptyp] and “humans”[MeSH Terms] and (English [lang] OR Japanese [lang]). For the search of Japan Medical Abstracts Society, the following combinations of search terms were used: (((pancreatitis / TH) AND (adrenal cortex hormone / TH)) and (PT = case report)) and (“pulse therapy (drug treatment)” / TH). We retrieved eight relevant cases of acute pancreatitis (2 from MEDLINE® and 6 from the database of Japan Medical Abstracts Society). The synopses of the cases including the present case are summarized in Table 2.

**Table 2:** Previously reported cases and the present case of acute pancreatitis developing after administration of corticosteroid pulse therapy

References (year)	Age (yr) / Sex	Primary Disease	Cardinal symptoms	Doses of steroids	Peak serum amylase (U/L)	Period for treatment at the episode (day)	Concomitant drugs	Outcome
The present case	51/F	Autoimmune hepatitis	Back pain	mPSL 1000 mg/day, then PSL 60 mg/day	436	5	Famotidine	Survived
Nishimoto et al. [20] (2014)	18/M	Acute monocytic leukemia	Abdominal pain	mPSL 1000 mg/day	451	5	Cytarabine, imipenem/cilastatin, piperacillin/tazobactam, vancomycin, meropenem	Survived
Jimi et al. [21] (2013)	70/F	ANCA-associated nephritis	Epigastric pain	mPSL 1000 mg/day, then PSL 35 mg/day	2400	5	NA	Survived
Suganuma et al. [22] (2012)	2/M	Kawasaki Disease	Abdominal pain	mPSL 15 mg/kg/day (BW, 13.5 kg), then PSL 10 mg/day for 3 days, then 5 mg/day for 3 days	2075	7	Flurbiprofen	Survived
Iwata et al. [23] (2010)	9/F	MPO-ANCA-associated glomerulonephritis	Abdominal pain	mPSL 30 mg/kg/day (BW, 22.8 kg), then PSL 25 mg/day	159	5	Mizoribine	Survived
Tsuruoka et al. [24] (2008)	72/F	ANCA-associated glomerulonephritis	Back pain, epigastric pain	mPSL 500 mg/day, then PSL 20 mg/day	306	20	NA	Died
Nakayama et al. [25] (2004)	60/F	MPO-ANCA-associated glomerulonephritis	Epigastric pain	mPSL 500 mg/day, then PSL 40 mg/day	1519	5	Cyclophosphamide	Survived
Kotaka et al. [26] (2002)	67/F	Myasthenia gravis	Back pain, vomiting	mPSL 1 g/day	3593	2	NA	Survived
Yoshizawa et al. [7] (1999)	80/F	Bullous pemphigoid	Nausea, epigastric pain	mPSL 1 g/day, then PSL 30 mg/day	1195	4	Alfacalcidol, calcium lactate, propentofylline, vinpocetine, omeprazole	Survived

*NA* no information was available in original report, *mPSL* methylprednisolone, *PSL* prednisolone, *BW* body weight

## Discussion

We believe that the present case report provides another line of evidence supporting the causality between the administration of corticosteroid pulse therapy and the development of acute pancreatitis. In the present case, the diagnosis of acute pancreatitis was definite, since it was made not only based on clinical symptoms and biochemical data but was confirmed by CT images. In contrast, the causal relationship between the administration of corticosteroid and the development of acute pancreatitis can only be inferred by exclusion of other possible causes. Acute pancreatitis has been reported to be associated with diverse clinical conditions such as cholelithiasis, heavy alcohol consumption, hypertriglyceridemia, hypercalcemia, pancreatic divisum, systemic vasculitis such as SLE, polyarteritis nodosa, trauma, viral infection (such as mumps) and drug use. While our patient had a medical history of cholelithiasis and gallbladder polyps, she underwent cholecystectomy one year earlier and the possibility of recurrent cholelithiasis or malignancy at the hepato-pancreatic region was excluded by the findings obtained from repeated CT scan examinations. In addition, she had never consumed alcohol or smoked before developing acute pancreatitis. While she took famotidine 40 mg/day during the corticosteroid pulse therapy, a previous nested case-control study performed in UK reported no cases of idiopathic pancreatitis among current users of famotidine [6]. Previous studies suggested a dose-dependent risk of developing pancreatitis during corticosteroid treatment, with thresholds of 25 mg/day for prednisolone [7]. In this context, patients receiving corticosteroid pulse therapy may have a higher risk of developing pancreatitis than those receiving lower doses of corticosteroids. In addition, a previous population based-cohort study suggested that acute pancreatitis developed between 4 and 14 days after the initiation of corticosteroids [8]. Our literature survey showed that 87.5% of the cases (7 out of 8 cases) developed pancreatitis within 14 days after the initiation of corticosteroids. In our case, pancreatitis developed 7 days after the initiation of steroid therapy. Collectively, we consider that the development of acute pancreatitis in the present case was most likely due to the methylprednisolone pulse therapy.

Many cases of acute pancreatitis in patients receiving corticosteroids combined with other drugs have been reported [9–14]. However, the causality between the administration of corticosteroids and development of acute pancreatitis in these cases remains inconclusive, since the patients treated with corticosteroids were diagnosed with autoimmune diseases manifesting systemic vasculitis (such as SLE and polyarthritis nodosa), which are sometimes complicated with pancreatitis irrespective of the administration of corticosteroids. Patients with antineutrophil cytoplasmic antibody (ANCA)-associated glomerulonephritis have been reported to be complicated with acute pancreatitis [15–17]. In addition, patients affected by autoimmune diseases receive corticosteroids as well as immunosuppressive drugs (such as azathioprine) that are associated with high risk of developing pancreatitis. Our literature survey revealed that many cases of acute pancreatitis developing after administration of corticosteroid pulse therapy were diagnosed with systemic vasculitis (such as ANCA-associated glomerulonephritis) or were given pancreatitis-inducing drugs (such as cyclophosphamide). In contrast, the present case had primary diseases that are unlikely to be associated with pancreatitis and received no drugs that may induce pancreatitis. Collectively, we considered that administration of methylprednisolone pulse therapy rather than the primary disease was responsible for the pancreatitis. Based on this clinical judgement, we withdrew prednisolone using a short-term tapering method. No signs of exacerbation in AIH activity were detected, probably because corticosteroids are highly effective in suppressing the disease activity of AIH. Previous studies showed that corticosteroids were effective in 36 to 100% [18] and 92% [19] of AIH patients.

## Conclusions

The present case and some reported cases strongly suggest a causal relationship between the administration of corticosteroids and the development of acute pancreatitis.

## Abbreviations

AIH: Autoimmune hepatitis
ANCA: Antineutrophil cytoplasmic antibody
SLE: Systemic lupus erythematosus
